# Correlation challenges for North Sea offshore wind power: a Norwegian case study

**DOI:** 10.1038/s41598-023-45829-2

**Published:** 2023-10-31

**Authors:** Martin Hjelmeland, Jonas Kristiansen Nøland

**Affiliations:** 1 Unaffiliated, Kastanjevegen 14, 4051 Sola, Norway; 2 Department of Electric Energy, Norwegian University of Science and Technology (NTNU), O. S. Bragstads plass 2E, 7034 Trondheim, Norway

**Keywords:** Energy grids and networks, Electrical and electronic engineering, Energy infrastructure, Renewable energy, Wind energy, Energy storage

## Abstract

Offshore wind power projects are currently booming around the North Sea. However, there are inherent correlation challenges between wind farms in this area, which has implications for the optimal composition of locations and the scale-up of installed capacities. This paper is aimed at addressing the correlation problem by minimizing the variance of total wind power accumulated around the North Sea. We show that this nonlinear convex optimization problem can be solved by applying the Augmented Lagrangian Algorithm (ALA). The premise of the study is that more interconnections between the EU countries will be prioritized in order to optimize and smooth out the wind power production patterns. A publicly available dataset with historical hour-by-hour data spanning over 20 years was used for the analysis. We explore two distinct scenarios for Norwegian offshore wind development. In the first scenario, we consider the ongoing activities on the European continental side of the North Sea and their implications for Norway. Here, we illustrate the advantages of focusing on expanding wind power capacity in the northern regions of Norway to enhance the overall value of the generated wind power. In contrast, the second reference scenario neglects these interconnections, resulting in a significantly greater concentration of offshore wind development in the southern parts of Norway, particularly in Sørlige Nordsjø II. Additionally, our work estimates the wind power correlation coefficient in the North Sea as a function of distance. Furthermore, we analyze deviations and intermittencies in North Sea wind power over various time intervals, emphasizing that the perceived integration challenges are highly dependent on the chosen time resolution in the analysis.

## Introduction

To curb climate change and reduce $\hbox {CO}_{{2}}$ emissions, countries around the North Sea are looking towards offshore wind power. The North Sea has a high potential for offshore wind development, with favorable wind conditions, shallow waters, and proximity to large markets. However, offshore wind power also faces some challenges related to its variability and integration into the grid. While the accelerated deployment of large clustered wind farms undeniably contributes to reduced greenhouse gas emissions, it’s imperative to recognize that such deployments can also alter local climate conditions and potentially reduce future resource potentials^1^. Consequently, it is imperative that future climate change impact studies consider these alterations^2^. Nevertheless, it is noteworthy that the wind resources in northern Europe do not appear to be significantly impacted by climate change^3^. Other environmental impacts may also be included, as currently, the scientific literature lacks detailed studies regarding the ecological risks of offshore wind power^4^.

The North Sea is adjacent to several countries in Northern Europe, and each country’s Exclusive Economic Zone (EEZ) defines the available wind resources^5^. There are several initiatives underway to coordinate the development of offshore wind around the North Sea. For instance, the North Sea Energy Cooperation (NSEC) by the European Commission supports and facilitates the development of offshore grid development and the large potential for renewable energy in the region^6^. The group includes countries like the Netherlands, Belgium, Germany, Denmark, Norway, and Sweden. Another ongoing project is the North Sea Wind Power Hub (NSWPH) that aim to ensure that offshore wind development does not become fragmented, country by country, but transnational and coordinated^7^. A core concept is to build a large artificial island in the North Sea that would serve as a hub for connecting offshore wind farms in the region. The project was founded by Transmission System Operators (TSOs) from Denmark, Germany, and Netherlands.

In 2022, energy ministers from Denmark, Belgium, Netherlands, and Germany came up with a joint declaration for offshore wind in the North Sea with the goal of substituting fossil fuels with renewables from the North Sea^8^. In addition, the Norwegian government has stated plans to grant licenses for 30GW offshore wind by 2040^9^. United Kingdom (UK) has also targets for offshore wind developments, with an ambitious target of a fivefold increase to 50GW in 2030^10^. Nevertheless, ambitious plans for offshore installations in the North Sea were already established in 2016^11^. At the time of writing this paper, there were plans for over 500GW of installed offshore wind around the North Sea, which is the main offshore region for Northern Europe^1^.

**Figure 1:**
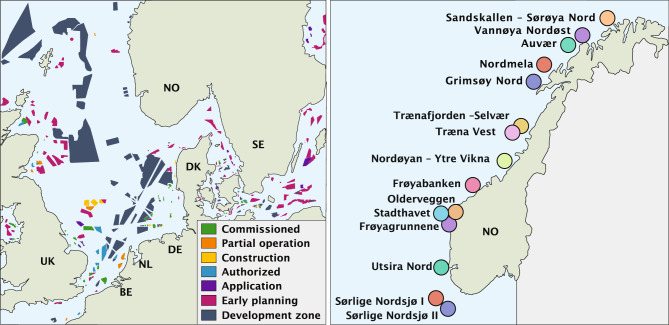
Map over the North Sea showing the status of the wind farms^12^ and map over the areas the Norwegian water resources and energy directorate (NVE) has pointed out as potential offshore wind areas.

**Table 1:** Overview of government targets for offshore wind deployments for the first half of 2022 taken from Global Wind Energy Council (GWEC)^13^.

Country	2022	2030	2035	2040	2045	2050
Norway	0 GW			30 GW		
Denmark	2.3 GW	10.0 GW				$\le$ 35.0 GW
Germany	7.7 GW	30.0 GW	40.0 GW		70.0 GW	
Belgium	2.3 GW	5.8 GW		8.0 GW		
Netherlands	3.0 GW	21.0 GW				
United Kingdom	13.6 GW			50.0 GW		
Cumulative total	28.9 GW	95.7 GW	135.7 GW	223.7 GW	293.7 GW	$\le$ 328.7 GW

Norway’s efforts to harness offshore wind power are well underway, with an 88MW floating wind farm, Hywind Tampen, officially launched in 2023. It is the world’s first floating wind farm to power offshore oil and gas platforms, providing electricity for the Norwegian North Sea’s Snorre and Gullfaks oil and gas fields. The estimated investment cost of Hywind Tampen was $691 million or $7.9 million per MW, with the Norwegian authorities pledging up to $262 million via Enova subsidies. In addition, the Norwegian business sector’s NOx fund offered up to $64 million. The wind farm is expected to meet about 35% of the electricity demand of the two fields. This will cut CO_2_ emissions from the fields by about 200,000 tonnes annually.

Nonetheless, as ambitious as these offshore wind development plans may be, they face significant challenges. Studies indicate that the wind power sectors in countries such as France, Germany, and Denmark will require continued subsidies to remain profitable^14^. Although costs associated with onshore and offshore wind power have declined over recent decades^15^, the downward trend ceased in 2020, with prices returning to 2015 levels by early 2023. Supply chain disruptions and rising energy and interest costs have been identified as potential factors influencing this shift^16^.

To understand the impact that larger shares of intermittent energy have on the grid, we may introduce the cannibalization effect and its influence on the capture price obtained by intermittent energy sources^17^. In essence, the cannibalization effect suggests decreasing power prices when a substantial amount of energy is supplied simultaneously. As a result, the capture price obtained by these power plants could be lower than that obtained by baseload power plants, which can operate continuously.

Reducing the correlation between various offshore wind farms can lessen the overall power variability across the entire fleet. Enhanced stability in power output, in turn, increases the chances of generating electricity during peak market prices, thus leading to a higher capture rate^18^. Consider, for instance, that strategic placement of these wind farms might lift the capture rate from 80 to 85%. For a hypothetical 30 GW Norwegian offshore wind power fleet, this difference is significant. With a 50% capacity factor and a baseload power price of $60/MWh, this 5% boost in the capture rate could result in $394 million in additional annual revenues, equivalent to $3/MWh. This scenario highlights the importance and potential financial benefits of smart placement and operation strategies.

In light of these considerations, this paper focuses on exploring the correlation challenges related to offshore wind power in the North Sea to evaluate the potential for increasing its value, with a focus on the Norwegian case. As shown in Fig. 1, we discuss the deployment activities and plans for the North Sea and the nve locations considered for Norway. A summary of the offshore wind targets and their installed capacity by 2022 is provided in Table 1. The considered nve locations for Norwegian offshore wind are described in Table 2.

**Table 2:** Overview of all the geographical areas pointed out as offshore wind power areas by the Norwegian coast.

Geographical area	Sea area (km$^2$)	Capacity factor (%)	Assumed buildout ^19^ (GW)	Min–mean–max depth below surface (m)	Min–max distance from shore (km)
Sandskallen - Sørøya nord	260	48.8	0.9	23–54–70	14–28
Vannøya Nordøst	154	43.8	0.5	5–43–70	0–9
Auvær	105	42.8	0.4	5–33–70	11–21
Nordmela	332	36.7	1.1	5–49–65	2–18
Gimsøy Nord	245	43.8	0.9	5–29–70	1–14
Trænafjorden - Selvær	197	41.4	0.7	5–32–70	26–40
Træna Vest	773	49.6	2.7	181–271–352	45–62
Nordøyan - Ytre Vikna	140	47.5	0.5	5–37–70	12–17
Frøyabanken	819	48.0	2.8	160–210–314	34–60
Stadthavet	520	57.5	1.8	168–208–264	58–84
Olderveggen	76	43.1	0.3	6–43–70	2–9
Frøyagrunnene	58	49.1	0.2	6–33–70	9–18
Utsira Nord	1010	51.4	3.5	185–267–280	22–53
Sørlige Nordsjø I	1375	58.8	4.8	50–64–70	149–209
Sørlige Nordsjø II	2591	59.3	9.0	53–60–70	140–214

Existing research already acknowledge that several challenges associated with offshore wind energy need to be addressed. These challenges include extreme weather conditions like *Dunkelflaute*, which can result in prolonged reductions in wind power generation^20^. To tackle these power fluctuations, one proposed solution for countries like Poland involves the construction of smaller, geographically dispersed wind farms^21^. Additionally, research has delved into the intermittency of wind power and potential mitigation strategies, often examining hypothetical electricity grids. Findings indicate that connecting wind farms across larger geographic areas can help smooth out some of the variability in wind power generation^22^. Studies on North Sea offshore wind power variability have been conducted, specifically focusing on regions such as the UK^23^. Moreover, the concept of complementarity between wind and solar power has been suggested as a potential hybrid energy system solution to reduce seasonal variability, though its effectiveness varies depending on the temporal scale^24,25^. Another innovative approach involves co-locating offshore wind and wave power to mitigate variability in individual renewable energy sources^26^. The role of diurnal smoothing, influenced by spatial variations in wind speed timing, has also been explored^27^. Moreover, there are several proposals for producing hydrogen as an ancillary service to address offshore wind power variability and facilitate further expansion both from researchers^28,29^. Furthermore, a hybrid hydrogen-battery storage system has been presented as a promising solution^30^. Alternative approaches, such as demand flexibility measures, have been proposed in lieu of strategically distributing wind farms^31^. Another alternative is to expand firm, dispatchable generation capacities, such as hydropower^32^, although it’s important to note that the potential for this option is limited in many regions worldwide. This paper aims to contribute to the optimization of offshore wind farm placement by introducing a method to reduce overall correlation among buildouts, thus enhancing geographical smoothing. This paper focuses on a particular research gap that has been identified. A comprehensive analysis of the interactions between different sites within a country and its interactions with other North Sea producers is currently missing. We aim to fill that gap with a focused case study for Norway. The primary contributions of this paper include:

1. A correlation analysis of offshore wind power in Northern Europe; and,
2. A methodology to minimize the variance of a wind power fleet using the Augmented Lagrangian Algorithm (ALA), which has the benefit of obtaining the unique optimal solution to nonlinear, convex optimization problems.

We intend to demonstrate that a weighted combination of the proposed Norwegian offshore wind farm locations ensures the least intermittent power output. This approach is fundamentally different from focusing on building out in locations with the best resources and being less concerned about the variability. While this study primarily explores offshore wind buildout optimization in the Norwegian North Sea, related research has examined the consequences of the assumed buildouts^19^. Additionally, a recent work has investigated the potential for strategically distributing wind farms in Brazil to significantly mitigate seasonal variability in offshore wind power using a genetic algorithm (GA)^33^. Given the nonlinear and convex nature of our optimization problem, we have determined that our proposed ALA is a more suitable choice than GA. Some optimization strategies have focused on enhancing individual wind farm performance or optimizing annual energy output^34^. Recent efforts have also concentrated on refining the methodology for estimating and validating wind resources and variability in the North Sea and Northern Europe^35,36^. Marine spatial planning (MSP) represents another avenue for coordinating offshore wind buildouts with other marine activities, thereby maximizing synergies with offshore energy generation^37^.

The rest of the paper is structured as follows: the ’Methodology and assumptions’ section details the proposed methodology to minimize the variance of a wind power fleet and describes the data collection process. The ’Results and discussion’ section presents and discusses our findings, and finally, the ’Conclusions’ section summarises the study’s key insights.

## Methodology and assumptions

This section focuses on the methodological approach undertaken in this study. It first introduces a proposed algorithm designed to minimize the variance of a wind power fleet’s power outputs. This addresses the challenge of correlation, a major factor in the variability of wind power generation. The algorithm’s design allows it to handle and balance power outputs from various wind farms. Finally, a description of the dataset and case studies used to apply and test the efficacy of this algorithm is provided.

### Optimization algorithm

In order to solve the correlation problem, the idea is to leverage power outputs to reduce variance and boost the system-level capacity factor rather than maximizing overall energy output. To minimize the variance of the sum of the weighted power outputs from the different farms, the weights need to be defined in an optimization variable (vector) $\textbf{x}\in {\mathbb {R}}^{N}_{+}$, where

1 $$\begin{aligned} \textbf{x} = \begin{bmatrix} x_1 &{} x_2 &{} x_3 &{}... &{} x_i &{}... &{} x_N \\ \end{bmatrix}. \end{aligned}$$

We define the matrix, $\textbf{Y} \in {\mathbb {R}}^{N\times T}$ in Eq. (2), where a row represents power output for an offshore wind farm $i\in {\mathbb {R}}^{N}$ over each time step in the time horizon $t\in {\mathbb {R}}^{T}$.

2 $$\begin{aligned} \textbf{Y} = \underbrace{\begin{bmatrix} y_{11} &{} y_{12} &{} y_{13} &{}... &{} y_{1t} &{}... &{} y_{1T} \\ ... &{}... &{}... &{}... &{}... &{}... &{}... \\ y_{i1} &{} y_{i2} &{} y_{i3} &{}... &{} y_{it} &{}... &{} y_{iT} \\ ... &{}... &{}... &{}... &{}... &{}... &{}... \\ y_{N1} &{} y_{N2} &{} y_{N3} &{}... &{} y_{Nt} &{}... &{} y_{NT} \end{bmatrix}}_{\text {Time horizon}} \end{aligned}$$

To leverage the opportunity to either minimize the variance or maximize the capacity factor, the objection function to be minimized is defined as,

3 $$\begin{aligned} f(\textbf{x}) = -\alpha \cdot \textrm{Mean}(\textbf{xY}) + \big (1-\alpha \big )\cdot \textrm{Var}(\textbf{xY}), \end{aligned}$$

where $\alpha \in [0,1]$ is a predetermined parameter. The problem is constrained such that the sum of the weights equals one, $\sum ^N_i x_i = 1$, and weights have to be non-negative, $x\ge 0$. By setting $\alpha =0$ and solving, i.e. minimizing only the variance of the weighted sum of some variables, we get a result that reduces both the variance of the variables and the covariance between them. As seen from the definition of variance between two variables *a* and *b*; $\textrm{Var}(a+b)=\textrm{Var}(a) + \textrm{Var}(b) + 2\textrm{Cov}(a,b)$. To simplify our problem, we reformulate our problem in the following general form

4a $$\begin{aligned}&\underset{x}{\min }\quad \qquad \quad \qquad \qquad \qquad f(\textbf{x}), \end{aligned}$$

4b $$\begin{aligned}&\text {subject to} \qquad \quad \quad \quad \qquad c(\textbf{x}) = 0, \end{aligned}$$

4c $$\begin{aligned}&\qquad \qquad \qquad \qquad \qquad \qquad h(\textbf{x}) \le 0. \end{aligned}$$

Our problem Eq. (4) is a nonlinear convex optimization problem. To solve it, we apply the Augmented Lagrangian optimization algorithm^38^, a widely used method for solving constrained optimization problems. Since the problem has a convex objective function and constraints, the algorithm will converge to a globally optimal solution, given that its penalty parameter $\rho$ is chosen appropriately. The Augmented Lagrangian of Eq. (4) is defined as

5 $$\begin{aligned} \begin{aligned} {\mathcal {L}}_{\rho }(\textbf{x}, \lambda , \mu , \rho ) = f(\textbf{x}) + \lambda ^T c(\textbf{x}) + \mu ^T h(\textbf{x}) + \frac{\rho }{2} c(\textbf{x})^T c(\textbf{x}) + \frac{1}{2}h(\textbf{x})^TI_{\rho } h(\textbf{x}), \end{aligned} \end{aligned}$$

where $\lambda$ and $\mu$ are the Langrangian multipliers associated with Eqs. (4b) and (4c), respectively. The identity matrix $I_{\rho }$ has zero valued diagonal elements for index *i* if $h_i(\textbf{x})<0$ and $\mu _i=0$, else the diagonal values are $\rho$. The algorithm for solving the Augmented Lagrangian problem is given by Algorithm 1 (The code for the project can be found at: https://github.com/martinhjel/wind-covariation).

**Algorithm 1:**
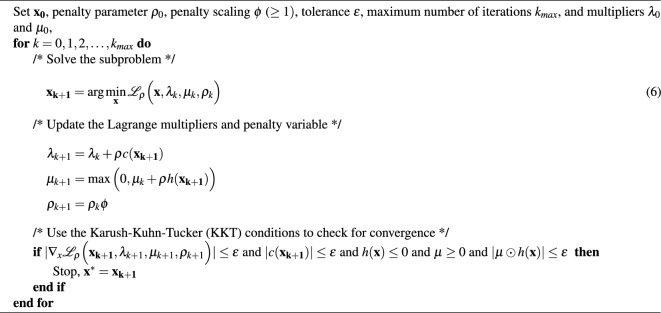
Augmented Lagrangian Algorithm.

By constructing and applying the Augmented Lagrangian optimization algorithm, we are setting the groundwork for a practical solution to the problem of power intermittency in offshore wind farms. Thus, the optimization algorithm is an instrumental tool in this research, providing a practical, mathematical framework to minimize the variance of a wind power fleet, leveraged with its capacity factor.

**Figure 2:**
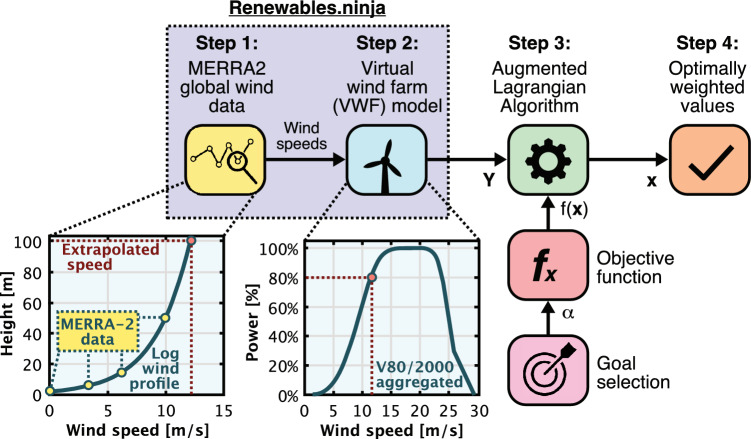
Methodological flowchart outlining the main steps taken in this study.

### Data collection

In this study, we rely on data collected from renewable.ninja^39^ that is based on NASA’s Modern-Era Retrospective analysis for Research and Applications (MERRA)^40^. MERRA is a reanalysis product that provides a consistent, long-term record of global weather patterns, which makes it a valuable tool for assessing wind resources. MERRA uses an advanced data assimilation system to integrate various observational data, including satellite measurements, into a global weather model, resulting in a comprehensive dataset of wind speed and direction, among other meteorological variables, at multiple altitudes and time intervals. The data has a spatial resolution of 0.5 degrees, hourly time resolution, and spans from 1979 to present time. The data provided by renewable.ninja and used in this work, spans from 2000 to 2019.

Despite the strengths of MERRA, like any reanalysis product, it is not without its limitations. To address these and enhance the accuracy of the wind resource estimates, renewable.ninja apply national correction factors. These factors adjust for systematic biases in the MERRA data and help align it more closely with nationally observed wind conditions, yielding an $\hbox {R}^2$ value above 0.95^41^. The hourly power production is calculated via Renewable Ninja’s virtual wind farm (VWF) model^42^, based on hourly wind speed and temperature data from NASA’s MERRA and MERRA-2 reanalysis, which has been validated for the UK^43,44^. Furthermore, the VWF was evaluated against actual power outputs from the Smøla (150MW) onshore wind farm in Norway (with Gaussian filtering) and for the Hywind demo (2.3MW) offshore flowing wind turbine in the Norwegian North Sea (without any Gaussian filtering)^19^. While there may be some uncertainty in the mean power output (i.e. capacity factors), it is still deemed that the production variability from hour to hour is well represented.

As highlighted in step 1 in Fig. 2, the VWF collects wind speeds at 2, 10, and 50 meters above ground at each spatial position of the MERRA grid dataset. Extrapolation of the wind speed at the turbine’s height above sea level (100 meters) is based on the logarithmic profile law. Working out the exact wind speed from reanalysis data for a specific turbine height is computationally expensive. Finally, the VWF model converts estimated wind speeds to power outputs using a real-world wind turbine power curve (i.e. Vestas V80/2000), which are aggregated to represent a complete wind farm better. This is done using a Gaussian filter with standard deviation ($\sigma = 0.2$) for the single wind turbine power curve. Assuming one specific power profile is a limitation, as there is a possibility to derate or redesign the turbine, at some higher cost, to produce relatively more at lower wind speeds. However, we assume in this study that the most cost-effective wind turbines will be prioritized offshore. Since wind turbine power curves exhibit relatively uniform characteristics, the specific selection of a power curve holds limited significance within the scope of the current study.

When compared with other reanalysis datasets, such as the European Centre for Medium-Range Weather Forecasts (ECMWF) Re-Analysis (ERA5) or the National Centers for Environmental Prediction (NCEP) North American Regional Reanalysis (NARR), MERRA exhibits some beneficial features. For one, it is widely recognized for its high temporal resolution and its accuracy in complex terrain and coastal regions, making it particularly suitable for offshore wind resource assessment. However, it should also be noted that the accuracy of reanalysis products can vary depending on the geographical region and the specific weather variables of interest.

A study compared the performance of the fifth generation of the European Centre for Medium-Range Weather Forecasts Reanalysis (ERA5) reanalysis and the Modern-Era Retrospective analysis for Research and Applications, version 2 (MERRA-2) reanalysis in modeling wind power generation for five different countries and 1051 individual wind turbines in Sweden^45^. The results showed that ERA5 performs better than MERRA-2 in all analyzed aspects, with correlations higher and errors lower. This finding could indicate that the quality of the data used in this study could be improved by using multiple datasets and cross-validation for a comprehensive and reliable wind resource assessment. This is, however, deemed out of the scope of this study, and we encourage research on this to open source their datasets to aid future research.

The MERRA-2 dataset has been utilized in studying offshore wind over a 30-year period in various locations^46^. Other datasets include the NORA3-WP dataset, which encompasses the Baltic, North, Norwegian, and Barents Seas^47^. The NVE provides an alternative dataset^48^, which is influenced by work from^49,50^ and contains historical data for offshore wind regions pertinent to this study. However, for ease of modeling and data uniformity, this study solely utilizes the renewable ninja data.

Previous studies using NVE data have provided insightful findings. A 2020 report from NVE^51^ revealed that in years with low hydro inflow, temperatures are also cold and lower wind power outputs are observed. Norway, with its varying wind conditions between the north and south regions, experiences the highest wind power output during winter. Unfortunately, in the coldest winter days with the highest demand wind resources are scarce. The report also underlines the grid balancing challenges posed by rapid fluctuations in power outputs from wind and solar sources.

The data is collected by computing the centroids of the proposed Norwegian wind farms, and some selected regions from the other North Sea countries (see Fig. 10a) and querying the renewable ninja API.

## Results and discussion

This section presents and interprets the results of our study, which primarily leveraged data obtained from renewable.ninja. Through a careful assessment of these data, we were able to extract meaningful insights into offshore wind resource potential and variability. The data has been meticulously analyzed to produce a comprehensive set of findings, revealing significant patterns and trends. These findings not only contribute to our understanding of wind power potential but also shed light on the intricacies of wind behavior under varying geographical conditions.

In the following subsections, we present our results and discuss these findings. The objective is not just to present the raw data but to provide context and interpret the implications. We also discuss the strengths and limitations of our approach, providing an opportunity for further research to build upon our work.

While our findings do not encompass every potentiality due to the data and scope limitations, they form a strong basis for future research in this field and contribute valuable insights for stakeholders involved in wind power development.

The section is divided into two. The first part investigates the wind power data and correlation factors. Finally, the second part shows the result of the optimization algorithm for optimally sizing the different wind farms.

### Analysis of wind power data

**Figure 3:**
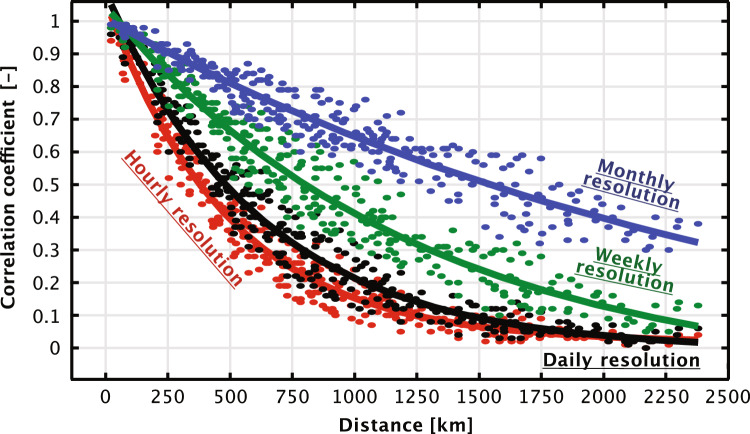
Scatter plot of correlation coefficients (*r*) between different wind farms with respect to distance and exponential curve fit of the data points. Results include hourly (1h), daily (24h), weekly (168h), and monthly (720h) time resolutions.

**Table 3:** Approximative exponential functions used to curve fit the correlation coefficient (*r*) data points in Fig. 3.

Hourly resolution	Daily resolution	Weekly resolution	Monthly resolution
$r \approx 1.05 e^{-\frac{1}{490.40d}} + 0.02$	$r \approx 1.10 e^{-\frac{1}{622.73d}} - 0.01$	$r \approx 1.15 e^{-\frac{1}{1242.21d}} - 0.1$	$r \approx 1.18 e^{-\frac{1}{2736.57d}} - 0.17$

The correlation coefficients between various wind farms around the North Sea have been gathered and graphically represented against the distance in Fig. 3 and compared with exponential curve fit functions described in Table 3. Exponential curve fits, corresponding to various resolutions including hourly (1h), daily (24h), weekly (168h), and monthly (720h), indicate that correlation diminishes as the distance between wind farms increases. It’s noteworthy that correlation coefficients drop below 0.5 at distances exceeding 384km at an hourly resolution and 808km at a weekly resolution. Higher time resolutions inherently smooth short-term fluctuations, thereby increasing correlation. However, during longer periods of wind drought, such as the one experienced in Europe in 2021 , the geographical dispersion of wind power farms becomes less effective, emphasizing the need for backup capacity and storage for longer durations.

The majority of correlation coefficients are positive, implying that negative correlation, which would be advantageous for risk diversification, is rare. Nevertheless, geographical dispersion of wind farms over long distances can significantly smoothen wind power output, but this effectiveness decreases over extended time periods. Given that the North Sea spans approximately 580 km in width and 960 km in length, wind farms established by individual countries will inevitably impact others. Thus, a coordinated approach toward the development of North Sea wind farms is vital for minimizing the overall variability of the region’s wind farm fleet.

**Figure 4:**
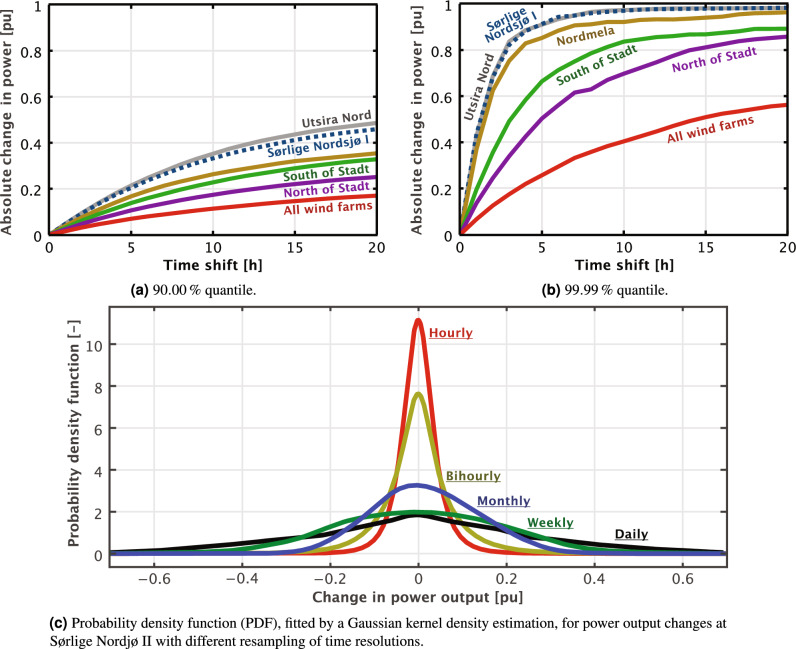
Changes in wind power output as a function of time with evenly distributed weights ($x_1$, $x_2$, ..., $x_{15}$ $=$ 6.67%).

**Figure 5:**
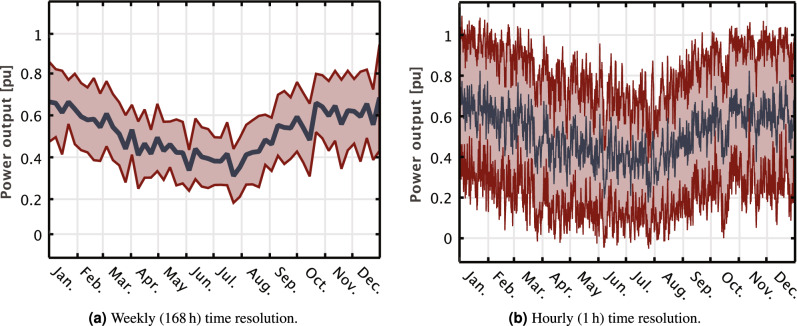
The yearly mean and standard deviation of wind power in the area of Utsira Nord for the years 2000 to 2019.

Malvaldi et. al.^52^ have already highlighted the decrease in correlation between wind farms with distance. They emphasized the need for prioritizing interconnections between EU countries to maximize wind power production and effectively manage its variability. This is further corroborated by Fig. 4, which reveals that the collective output from proposed Norwegian offshore wind farms exhibits far less variability than individual parks. Observe in Fig. 4a how, for the entire wind power fleet, it takes more than 20 hours for the absolute change to be more than 20% for 10% of the time (i.e. 90% quantile), while for 0.01% of the time (i.e. 99.99% quantile), in Fig. 4b, this change happens in just 4 hours. The probability for changes in power outputs for a single wind farm is shown in Fig. 4c, in which we can observe significant differences in behavior for different time scales.

Time resolution plays a pivotal role in evaluating the variability of wind power output, as illustrated in Fig. 4c. Higher variability in wind power output is evident at daily and weekly time resolutions, highlighting the necessity for backup capacity and energy during these periods. This may explain the rise of gas-fired power plants in Europe, as they are capable of fulfilling this role. Other researchers have also observed a need for increased backup energy and storage in wind-dominated power systems^53^. The successful integration of renewable energy, particularly wind power, has historically relied on the presence of fossil-based backup capacity^54^. However, reliance on coal power would lead to increased heat rates and, subsequently, increased fuel consumption, reducing the carbon mitigation effect by 13%^55^. Similar effects are also true for fast-acting single-cycle gas turbines compared to the more efficient but slow-acting, closed-cycle gas turbines. These findings underscore the importance of adopting a system-wide perspective for power system developments with regard to reducing CO_2_ emissions.

Figure 5 shows the variability of wind power from Utsira Nord for weekly and hourly time resolutions between different years. While variability is smoothed out over longer periods, Fig. 5b reveals significant standard deviation at the hourly resolution, emphasizing the need to combine outputs of several wind farms across a large geographical region. Furthermore, despite apparent seasonal trends that match well with the Norwegian demand profile, the availability of wind power cannot be guaranteed over shorter time scales, as the changes from hour to hour can be substantial.

**Figure 6:**
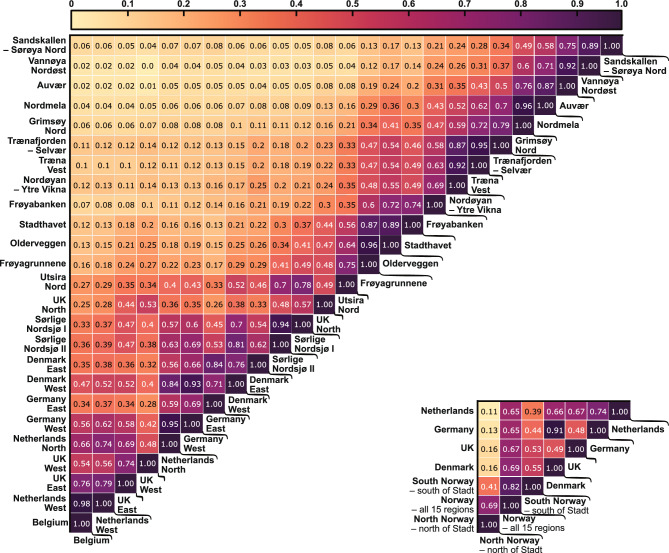
Upper left: Standard correlation coefficient matrix for all the studied regions. Bottom right: Standard correlation coefficient matrix for some aggregated areas. Coefficients calculated with a daily (24h) time resolution.

In Fig. 6, the wind power correlation relationships between the different wind power aggregated areas and locations are presented in matrix form, respectively. In the region of Stadt in Norway (the areas Stadthavet, Olderveggen, Frøyagrunnene), one can observe a drop in the correlation between the areas north and south. For instance, the correlation (*r*) from Sørlige Nordsjø II drops from 0.7 to 0.41 from Utsira Nord to Frøyagrunnene, and further to 0.19 to Frøyabanken. From these results, one could argue that in order to reduce the correlation with the rest of the North Sea countries and reduce the variance of the combined wind power fleet, Norway should focus on building out the northernmost areas. This hypothesis will be further evaluated when presenting the optimization results in the following subsections.

**Figure 7:**
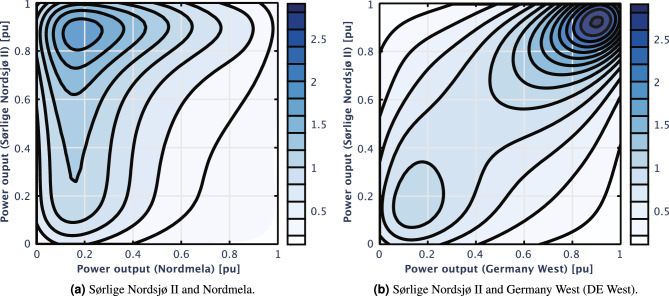
Bivariate distribution of wind power output by heatmaps generated using Gaussian kernel density estimation.

Further evidence illustrating how distance affects correlation is presented in Fig. 7a. The figure illustrates the bivariate distribution between Nordmela, a northern wind farm, and Sørlige Nordsjø II, the southernmost wind farm in Norway. It can be noted that when Nordmela has low power output, there’s a higher probability of Sørlige Nordsjø II displaying high power output.

In contrast, the bivariate distribution between Sørlige Nordsjø II and Germany West, as depicted in Fig. 7b, exhibits a more close connection. Notable observations include a significant peak distribution at high outputs for both farms and a smaller peak at low outputs. Evidently, the correlation between the southernmost Norwegian offshore wind farm, Sørlige Nordsjø II, and European regions, specifically Germany West, is substantial.

**Figure 8:**
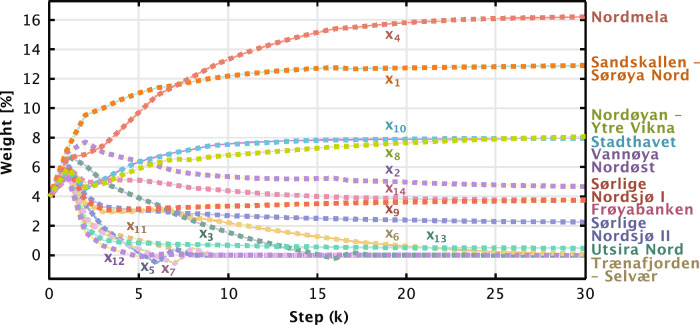
Trajectory of the learning of the weights for Norwegian offshore wind farms with hourly (1h) resolution, when the weights of the European offshore wind farms, $x_{16}$ to $x_{25}$, are fixed to 4%, and $\alpha = 0$.

**Figure 9:**
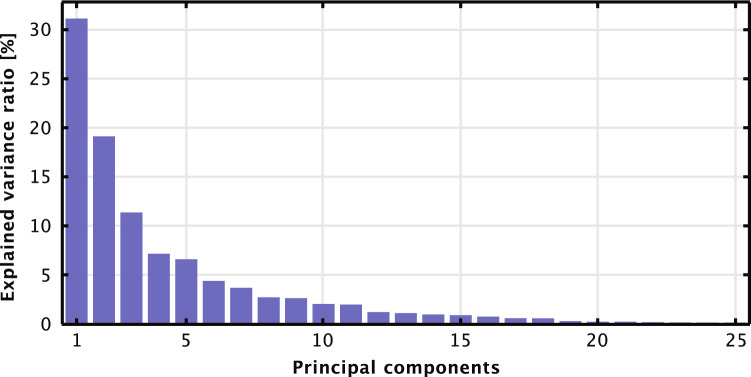
Principal component analysis (PCA) of the 25 studied regions.

To further examine the correlation patterns of the North Sea wind power data, we initiated a quantitative assessment of the dataset’s suitability for Principal Component Analysis (PCA) via the Kaiser-Meyer-Olkin (KMO) measure of sampling adequacy. A KMO value of 0.81 was determined, which denotes that a significant proportion of the variance in our variables is shared. From the PCA analysis in Fig. 9, we observed that out of 25 derived principal components, the predominant two jointly explained 50.3% of the variance in the dataset. The extensive variance elucidated by the primary two components accentuates the mutual patterns and correlations across the studied regions, providing a statistical underpinning to the visual and qualitative analyses.

It’s important to note that these findings are based on a model using a fixed turbine type. However, utilizing alternative turbine types, where lower energy output could be compensated with higher capacity factors, and thus more stable wind power conditions, may introduce a smoothing effect. This effect could in turn modify the correlation coefficients and decrease the variability of the wind power output. Evaluating these potential differences, though, is considered beyond the scope of the present study.

### Optimal weighting of offshore wind farms

This section employs the Augmented Lagrangian Algorithm to identify the minimum of the objective function defined in Eq. (3). Initially, the European offshore wind farm regions are each assigned a weight of 4%, implying that European sites account for 40% of the relative power system interacting with Norway. The algorithm commences with equal weights of 4% for all variables, as depicted in Fig. 8, and generally converges after several iterations. The rate of convergence is sensitive to the numerical tolerance accorded to the Karush-Kuhn-Tucker (KKT) conditions. For simplicity, we present only the first 30 steps.

Table 4 shows the converged values obtained in the learning process, alongside optimized values for scenarios with varying emphases on overall power output relative to the minimization of variance. Scenarios include those with $\alpha = 0.1$, $\alpha = 0.3$, and $\alpha = 0.5$ - the last of which equally weights overall power output and variance reduction. In this particular case, the Norwegian offshore wind power expansions occur solely in Stadthavet and Sørlige Nordsjø II, with the latter being highly weighted to deliver increased power output.

**Table 4:** Overview of the weighted outputs solving Eq. (3) with hourly (1h) resolution and different $\alpha$-weightings in the loss function using the optimization model. Norwegian wind farms are variable, while the European ones are fixed.

Geographical area	Variable	Optimized value (%)			
		$\varvec{\alpha = 0.5}$	$\varvec{\alpha = 0.3}$	$\varvec{\alpha = 0.1}$	$\varvec{\alpha = 0.0}$
Sandskallen - Sørøya Nord	$x_1$	0.00	**14.54**	** 17.85**	** 12.99**
Vannøya Nordøst	$x_2$	0.00	0.00	0.00	**4.52**
Auvær	$x_3$	0.00	0.00	**5.31**	0.00
Nordmela	$x_4$	0.00	0.00	0.00	**16.31**
Gimsøy Nord	$x_5$	0.00	0.00	**1.55**	0.00
Trænafjorden - Selvær	$x_6$	0.00	0.00	0.00	0.00
Træna Vest	$x_7$	0.00	**2.35**	**6.67**	0.00
Nordøyan - Ytre Vikna	$x_8$	0.00	0.00	**3.30**	**8.10**
Frøyabanken	$x_9$	0.00	0.00	0.00	**3.67**
Stadthavet	$x_{10}$	**28.27**	**23.07**	**14.62**	**7.96**
Olderveggen	$x_{11}$	0.00	0.00	0.00	0.00
Frøyagrunnene	$x_{12}$	0.00	0.00	0.00	0.00
Utsira Nord	$x_{13}$	0.00	0.00	0.00	**0.44**
Sørlige Nordsjø I	$x_{14}$	0.00	0.00	**2.93**	**3.87**
Sørlige Nordsjø II	$x_{15}$	**31.73**	**20.04**	**7.77**	**2.14**
UK North	$x_{16}$	** 4.00**	** 4.00**	** 4.00**	** 4.00**
Denmark East	$x_{17}$	** 4.00**	** 4.00**	** 4.00**	** 4.00**
Denmark West	$x_{18}$	** 4.00**	** 4.00**	** 4.00**	** 4.00**
Germany East	$x_{19}$	** 4.00**	** 4.00**	** 4.00**	** 4.00**
Germany West	$x_{20}$	** 4.00**	** 4.00**	** 4.00**	** 4.00**
Netherlands North	$x_{21}$	** 4.00**	** 4.00**	** 4.00**	** 4.00**
UK West	$x_{22}$	** 4.00**	** 4.00**	** 4.00**	** 4.00**
UK East	$x_{23}$	** 4.00**	** 4.00**	** 4.00**	** 4.00**
Netherlands West	$x_{24}$	** 4.00**	** 4.00**	** 4.00**	** 4.00**
Belgium	$x_{25}$	** 4.00**	** 4.00**	** 4.00**	** 4.00**
Sum of weights	$\sum {x_i}$	100.00	100.00	100.00	100.00
Mean power output	$\textrm{Mean}(\textbf{xY})$	55.93	54.27	52.14	49.12
Standard deviation	$\sqrt{\textrm{Var}(\textbf{xY})}$	± 20.82	± 18.06	± 16.60	± 16.16

The mean power output and standard deviation indicate the trade-off between maximizing expected output and minimizing its variance.

Significant values are in bold.

With the optimized weights in Table 5, the proportions of the European offshore wind farm regions can also be optimized. Notably, if we aim only to minimize variance, Denmark West, Germany West, Netherlands North, and Netherlands West will not see any buildouts. However, if overall power output becomes more significant, Germany West and Netherlands North play a larger role in the energy mix.

**Figure 10:**
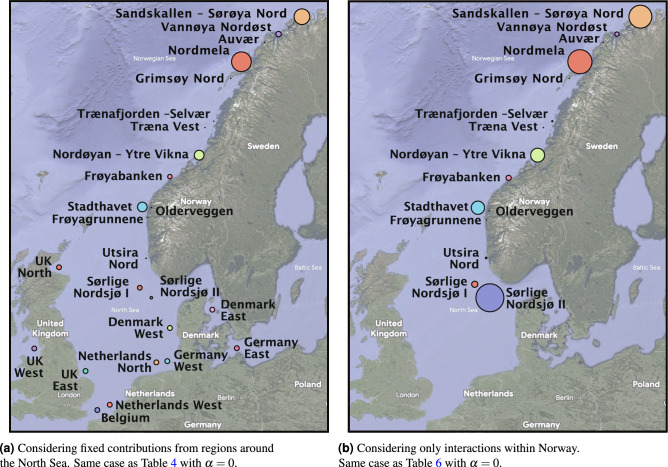
The scale of the dots for each area represents how offshore wind power should be built out to reduce the variance of the wind power fleet as a whole with hourly (1h) resolution. Note how the model gives little weight to the Norwegian offshore wind farms in the south (especially Sørlige Nordsjø II) when including wind farms for all countries around the North Sea.

Table 6 showcases optimized results for Norwegian offshore wind buildouts without considering European interaction. Here, if the objective is to minimize variance, less than a third of the buildouts will occur in southern Norway. Table 6 also shows that the assumed buildouts account for the majority in the South, yielding a higher overall variance. These results are in line with another study, where we have included their weightings of the wind farms in the table^19^. Figure 10 presents a map of relative buildouts considering and disregarding European interconnections.

Tables 4, 5, and 6 collectively indicate a tradeoff between maximizing wind power output and reducing correlation. This implies a need for strategic objective weighting, particularly when the ambition to diminish correlation competes with reducing the levelized cost of electricity (LCOE). Higher correlation can indeed lead to power price cannibalism, thereby diminishing revenues for the wind farms^18^.

**Table 5:** Overview of the weighted outputs solving Eq. (3) with hourly (1h) resolution and different $\alpha$-weightings in the loss function using the optimization model. All wind farms and regions are variable.

Geographical area	Variable	Optimized value (%)			
		$\varvec{\alpha = 0.5}$	$\varvec{\alpha = 0.3}$	$\varvec{\alpha = 0.1}$	$\varvec{\alpha = 0.0}$
Sandskallen - Sørøya Nord	$x_1$	0.00	** 15.87**	** 16.80**	** 10.84**
Vannøya Nordøst	$x_2$	0.00	0.00	0.00	** 5.07**
Auvær	$x_3$	0.00	0.00	** 5.11**	0.00
Nordmela	$x_4$	0.00	0.00	0.00	** 15.06**
Gimsøy Nord	$x_5$	0.00	0.00	** 1.06**	0.00
Trænafjorden - Selvær	$x_6$	0.00	0.00	0.00	0.00
Træna Vest	$x_7$	0.00	** 3.83**	** 7.09**	0.00
Nordøyan - Ytre Vikna	$x_8$	0.00	0.00	** 2.45**	** 6.71**
Frøyabanken	$x_9$	0.00	0.00	0.00	** 3.52**
Stadthavet	$x_{10}$	** 27.12**	** 20.47**	** 12.54**	** 6.52**
Olderveggen	$x_{11}$	0.00	0.00	0.00	0.00
Frøyagrunnene	$x_{12}$	0.00	0.00	0.00	0.00
Utsira Nord	$x_{13}$	0.00	0.00	0.00	** 0.10**
Sørlige Nordsjø I	$x_{14}$	0.00	0.00	0.00	** 0.29**
Sørlige Nordsjø II	$x_{15}$	** 42.00**	** 24.06**	** 11.55**	** 6.16**
UK North	$x_{16}$	** 24.31**	** 17.75**	** 10.30**	** 7.33**
Denmark East	$x_{17}$	0.00	0.00	0.00	** 1.56**
Denmark West	$x_{18}$	0.00	0.00	0.00	0.00
Germany East	$x_{19}$	0.00	0.00	** 11.03**	** 13.69**
Germany West	$x_{20}$	0.00	** 5.35**	** 2.18**	0.00
Netherlands North	$x_{21}$	** 6.57**	** 6.12**	** 1.23**	0.00
UK West	$x_{22}$	0.00	** 0.36**	** 6.53**	** 8.10**
UK East	$x_{23}$	0.00	** 6.19**	** 3.87**	** 0.58**
Netherlands West	$x_{24}$	0.00	0.00	0.00	0.00
Belgium	$x_{25}$	0.00	0.00	** 8.26**	** 14.47**
Sum of weights	$\sum {x_i}$	100.00	100.00	100.00	100.00
Mean power output	$\textrm{Mean}(\textbf{xY})$	58.18	55.77	52.06	48.40
Standard deviation	$\sqrt{\textrm{Var}(\textbf{xY})}$	± 22.71	± 18.77	± 16.30	± 15.73

The mean power output and standard deviation indicate the trade-off between maximizing expected output and minimizing its variance.

Significant values are in bold.

**Table 6:** Overview of the weighted outputs solving Eq. (3) with hourly (1h) resolution and different $\alpha$-weightings in the loss function using the optimization model. The interactions between only co-Norwegian wind farms are considered.

Geographical area	Variable	Optimized value (%)				Ref. ^19^ (%)
		$\varvec{\alpha = 0.5}$	$\varvec{\alpha = 0.3}$	$\varvec{\alpha = 0.1}$	$\varvec{\alpha = 0.0}$	
Sandskallen - Sørøya Nord	$x_1$	** 5.06**	** 21.65**	** 23.33**	** 18.96**	** 3.02**
Vannøya Nordøst	$x_2$	0.00	0.00	0.00	** 3.58**	** 1.78**
Auvær	$x_3$	0.00	0.00	** 6.36**	0.00	** 1.20**
Nordmela	$x_4$	0.00	0.00	0.00	** 20.00**	** 3.82**
Gimsøy Nord	$x_5$	0.00	0.00	** 4.07**	0.00	** 2.84**
Trænafjorden - Selvær	$x_6$	0.00	0.00	0.00	** 1.01**	** 2.26**
Træna Vest	$x_7$	0.00	** 7.29**	** 4.92**	0.00	** 8.95**
Nordøyan - Ytre Vikna	$x_8$	0.00	0.00	** 9.25**	** 11.50**	** 1.64**
Frøyabanken	$x_9$	0.00	0.00	0.00	** 4.32**	** 9.46**
Stadthavet	$x_{10}$	** 37.04**	** 27.56**	** 18.41**	** 10.98**	** 6.00**
Olderveggen	$x_{11}$	0.00	0.00	0.00	0.00	** 0.87**
Frøyagrunnene	$x_{12}$	0.00	0.00	0.00	0.00	** 0.66**
Utsira Nord	$x_{13}$	0.00	0.00	0.00	** 1.39**	** 11.68**
Sørlige Nordsjø I	$x_{14}$	0.00	** 1.70**	** 4.81**	** 5.09**	** 15.87**
Sørlige Nordsjø II	$x_{15}$	** 57.90**	** 41.80**	** 28.85**	** 23.17**	** 29.95**
Sum of weights	$\sum {x_i}$	100.00	100.00	100.00	100.00	100.00
Mean power output	$\textrm{Mean}(\textbf{xY})$	58.12	55.83	53.26	49.88	53.36
Standard deviation	$\sqrt{\textrm{Var}(\textbf{xY})}$	± 23.85	± 20.29	± 18.79	± 18.32	± 21.15

Comparison is made against the reference values of the assumed capacity distribution^19^. The mean power output and standard deviation indicate the trade-off between maximizing expected output and minimizing its variance.

Significant values are in bold.

Our optimization results indicate that prioritizing the development of the wind resource areas farthest away is key to reducing wind power variance around the North Sea. As there is a lot of activity on the European continental side of the North Sea^8^, it would be desirable to find ways to reduce the overall wind power correlation. Building a significant portion of Norwegian offshore wind capacity in the northern regions of Norway aligns with our optimization model, especially in terms of reduced correlation. However, this poses a dilemma, as these remote locations are also the costliest to develop. In a scenario where one accepts a higher wind power correlation, there will be more sporadic energy scarcity and abundance, and more of the aforementioned backup capacity will be required. However, suppose one aims to deal with the correlation problem with extensive energy storage solutions like battery storage. Here, predictions indicate that by 2031, Europe will only be capable of storing about 10 minutes of its electricity in batteries (i.e. 89GWh)^56^, which takes into account a twenty-fold increase from today’s levels. Nevertheless, Norwegian hydropower, with about half of Europe’s storage capacity (i.e. 87TWh)^32^, can play a role. However, the hydropower fleet’s dispatchable power capacity is limited to just 30GW. This underlines the importance of other flexible non-polluting actors in the energy system. Construction of a diverse power infrastructure could be beneficial to mitigate potential energy scarcity in Europe, considering other non-polluting energy sources like nuclear energy, as demonstrated by the Polish nuclear program^57^.

Nevertheless, there are several possible solutions to address the correlation challenges for wind power in the North Sea. First of all, the development in the region should be done in cooperation with all member countries, as compared to the current situation where only some countries have found together. Cooperation could harmonize energy policies, regulations, and standardization. Best practices could be shared for all countries to avoid common pitfalls. Local bottlenecks can be alleviated by increasing the interconnection capacity and ensuring that countries around the North Sea are coupled more tightly together. This has the benefit of utilizing the smoothing effect observed by wind power over larger distances, as seen in Fig. 3. The downside, however, is that countries may give away some of their security of supply as it, without any additional investments in local backup capacity such as gas-fired power plants, more often rely on the energy coming from elsewhere.

Closer power grid coupling could also enable better sharing of energy storage solutions and demand-side responses. However, countries with significant energy storage, like Norway with its hydro reservoirs, may experience increased variability due to interconnected energy availability. This increased variability could result in hydro reservoirs being operated less flexibly as the future energy availability becomes more uncertain. As a consequence, operators may be more inclined to maintain their reservoir levels at the midpoint.

Another method to deal with the correlation challenges is to improve forecasting, reduce the uncertainty and variability in supply and demand, and enable better scheduling of generation and transmission capacities. Sharing of data between wind farms would be beneficial but perhaps hard to achieve in a competitive market environment. Forecasting wind power is, however, a challenging task for periods longer than a couple of days and not useful after almost one week^58^, so this would only aid in the shorter term.

The reduction in correlation between offshore wind farms can be advantageous as it potentially lessens the variation of the total power output. However, such an approach may lead to more dispersed build-outs, thereby escalating the construction costs and necessitating extended transmission grids.

Our study illustrates that while expanding the Northern offshore wind farms would indeed lead to decreased correlation, it might not be economically feasible. This claim is backed by the historical costs of transmission grids in Norway, which stand at approximately $1.2 million per km per GW^59^. Considering Norway’s length of 1748km, the cost of a transmission line across the country would approximate $2100 million per GW transmission. This cost has to be contrasted with the potential increase in the capture rate, which could result in savings annually of $394 million, by 5% increase capture rate, as explained in the introduction. The net present value of such a hypothetical improvement is $4600 million, assuming an economic lifetime of 25 years and a discount rate of 7%. Clearly, the financial viability of such a project needs to be studied in further detail. In addition, the LCOE costs associated with not building out wind power in the areas with the best resources but selecting regions to reduce the overall correlation would also be necessary to take into account. This assertion calls for more detailed studies to draw more definitive conclusions.

## Conclusions

This paper presents a comprehensive mapping of the optimal composition of wind farms in the Norwegian North Sea, with a primary focus on minimizing correlation challenges and reducing grid integration costs. One of the scenarios in our research is the projected expansion of interconnections among EU countries, which would allow for the smoothing of wind power generation. Given this scenario, it becomes preferable to construct the majority of offshore wind farm capacity in northern Norway. This preference is further emphasized in the mapping of wind power correlation coefficients between Norwegian and European offshore wind sites. Notably, we found that northernmost sites exhibit a significant decrease in correlation with Europe, mainly due to their geographical distance. In the alternative scenario explored, offshore wind expansion occurs independently of any coordination with interconnections. This results in a notably distinct development pattern, with a substantial concentration of wind farm installations in southern Norway. It is crucial to underscore that this approach leads to a greater variability in wind power output, as it lacks the geographical smoothing effect achieved through collaboration with neighboring countries in the North Sea. In summary, our research underscores the importance of geographical smoothing, a concept with potential relevance for numerous countries in the North Sea region and beyond.

While the winter season shows a trend of increased wind power resources, our analysis at an hourly resolution indicates a considerable standard deviation in wind power output over 20 years of collected data. Nevertheless, we observe a smoothing effect when considering a collective of wind farms, as it can diminish the absolute change in power output.

The present paper has certain limitations, including the utilization of publicly available MERRA2 weather reanalysis data for numerical analysis. Wind speeds were estimated vertically using extrapolation grounded in the logarithmic profile law. Additionally, the power output, as influenced by wind speed, was determined based on the characteristics of a specific wind turbine type, and Gaussian filtering was applied to account for the geographical dispersion of wind farms, which is a simplified approach. Nonetheless, it is important to note that earlier studies have demonstrated that the paper’s primary focus, which is variations and correlations in wind power output, is adequately captured^19^. Nevertheless, when it comes to modeling wind resources, particularly mean power output, it is acknowledged that this approach may not be as precise when compared to real-world data.

For future research, we suggest utilizing multiple datasets (including ERA5) and performing cross-validation studies to enhance the reliability of wind resource assessments. This approach would expand beyond the scope of this paper, which primarily focuses on capturing wind resource variability. There should also be an investigation into how consumption patterns in different regions around the North Sea could influence the optimal sizing of wind farms, taking into account a detailed energy system model that balances the value of a more dispersed wind farm fleet against additional transmission, construction, and maintenance costs.

Furthermore, exploring the utilization of various turbine types to maximize energy outputs while reducing the variability of the combined wind power fleet would provide valuable insights for decision-makers involved in the ongoing development of offshore wind power in the North Sea region. This research could ultimately contribute to more efficient and sustainable energy strategies, promoting the successful integration of renewable energy sources into the grid.

## Data Availability

This paper relies primarily on leveraged data obtained from the renewable.ninja^39^ environment, which is based on NASA’s MERRA^40^. Derived raw data supporting the findings of this study are available from the corresponding author J.K.N. upon reasonable request.
